# Clinical observation of autologous platelet rich fibrin assisted revascularization of mature permanent teeth

**DOI:** 10.1186/s13005-023-00350-9

**Published:** 2023-03-15

**Authors:** Zhaojun Wu, Yao Lin, Xuehong Xu, Zhiqun Chen, Yan Xiang, Lvli Yang, Wei Zhang, Suli Xiao, Xiaoling Chen

**Affiliations:** 1 Department of Endodontics, Stomatological Hospital of Xiamen Medical College, Huli District, No.1309, Lvling Road, Xiamen, 361008 Fujian China; 2 Xiamen Key Laboratory of Stomatological Disease Diagnosis and Treatment, Fujian, 361008 China

**Keywords:** Platelet rich fibrin, Mature permanent teeth, Revascularization

## Abstract

**Objective:**

To investigate the clinical observation of autologous platelet-rich fibrin (PRF) assisting the revascularization of mature permanent teeth.

**Methods:**

Twenty patients with mature permanent teeth were divided into experimental group and control group. The control group was treated with classic revascularization, and the experimental group was treated with PRF-assisted mature permanent tooth revascularization.

**Results:**

After treatment, the total effective rate of the experimental group (100.00%) was higher than that of the control group (50.00%); the thickness of the root canal wall of the experimental group was higher than that of the control group, and the crown root length was lower than that of the control group; The bite degree, chewing function, color, overall aesthetic score, and satisfaction rate of the patients were higher, and the difference was statistically significant (*P* < 0.05).

**Conclusion:**

Autologous PRF assists in revascularization of mature permanent teeth, which can achieve ideal results, and promote pulp regeneration.

## Introduction

As a fully developed tissue, permanent teeth are difficult to recover once damaged [1]. When permanent teeth are fully mature and their development stops, the blood supply to the pulp is insufficient and can only come from the narrow apical foramen [2]. Therefore, traditional root canal treatment is the most common treatment method for mature permanent teeth with carious pulp exposure [3, 4]. The purpose of randomized controlled trial (RCT) is to debridement, chemically and mechanically debride the root canal system, and finally to hermetically fill the root canal system with biocompatible material [5, 6]. However, the filling materials easily discolor the crown, which affects the aesthetics of the patient's teeth [7]. Moreover, in the treated root canal, long-term Ca(OH)_2_ filling will reduce the flexural resistance of the dentin [8]. Therefore, it is particularly critical to seek an ideal treatment for permanent dental disease. Pulp revascularization is a common method used clinically to treat pulp diseases of permanent teeth. Although pulp revascularization is currently the only clinically approved "Regenerative endodontic treatment (RET)" treatment strategy, it still cannot fully meet the three requirements: the elimination of symptoms and evidence of bony healing, increased root wall thickness and/or increased root length, and positive response to vitality testing [9]. How to further improve the effectiveness of regeneration is still a topic of interest. Dental pulp revascularization forms blood clots in the pulp canal, which provides scaffolds and growth factors. Compared with whole blood, platelet rich fibrin (PRF) theoretically provides higher concentrations of fibrin and growth factors with potentially better therapeutic effects [10]. A recently published meta-analysis indicated that compared with blood clots, PRF has a higher 1-year mean success rate for apical closure or reduction (85.2% vs 58.8%) and root lengthening (74.1% vs 64.1%) [11]. However, there is still a lack of conclusions with significant differences, and more clinical studies are needed to confirm the results. In order to further understand its mechanism of action, through comparative studies, the clinical effect of PRF pulp revascularization in the treatment of mature permanent teeth is analyzed. details as follows.

## Subjects and methods

### Inclusion and exclusion criteria

Inclusion criteria: (1) 18–30 years old; (2) Immature necrotic permanent teeth [12]: tooth development is in stage 7, 8 or 9 of Nolla staging. The Nolla staging method is as follows: stage 0: no dental follicle appears; stage 1: imaging of the dental follicle appears; stage 2: beginning of calcification of the tooth tip; stage 3: crown formation of 1/3; stage 4: crown formation of 2/3; 5 Stage: the crown is almost formed; stage 6: the crown is formed and the root begins to develop; stage 7: the root is formed 1/3; stage 8: the root is formed 2/3; stage 9: the root is almost formed, the apical foramen is not closed; 10 Stage: Tooth root formation, apical foramen closed; (3) Adult permanent teeth with mature roots but with absorption damage to the apex, and the diameter of the apical hole is greater than 1 mm; (4) A restorable tooth; (5) There is no need to leave space for the final post/core restoration; (6) Anterior teeth or premolars with single canal; (7) A cooperative and compliant patient; (8) Patients are not allergic to the drugs and antibiotics which needed to complete treatment; (9) No periodontal disease or root cracking.

Exclusion criteria: (1) patients with other serious organ diseases, such as cardiopulmonary, kidney and other major diseases; (2) patients with apical cyst; (3) patients with poor cooperation and those who quit the study halfway.

### General information

20 patients with mature permanent teeth treated in our hospital (may 2019 ~ may 2021) were randomly divided into control group and experimental group, with 10 cases in each group. The general data are shown in Table 1 below. There is no significant difference between the two groups (*p* > 0.05). The study protocol was approved by the Ethics Committee of our institution (No. KS20220606001), and it meets the ethical requirement of the Helsinki Declaration.

**Table 1:** Comparison of general data between the two groups

group	n	Gender (male / female)	Age (years)	Course of disease (weeks)	Follow up time (months)
experience group	10	**5/5**	24.50 ± 6.50	4.10 ± 1.37	21.00 ± 3.00
Control group	10	**7/3**	24.00 ± 6.00	3.89 ± 1.20	21.50 ± 2.50
Statistical value	-	*x*^*2*^ = 0.208	*t* = 0.179	*t* = 0.365	*t* = 0.405
*P*value	-	0.648	0.860	0.720	0.690

### Method

**Fig. 1:**
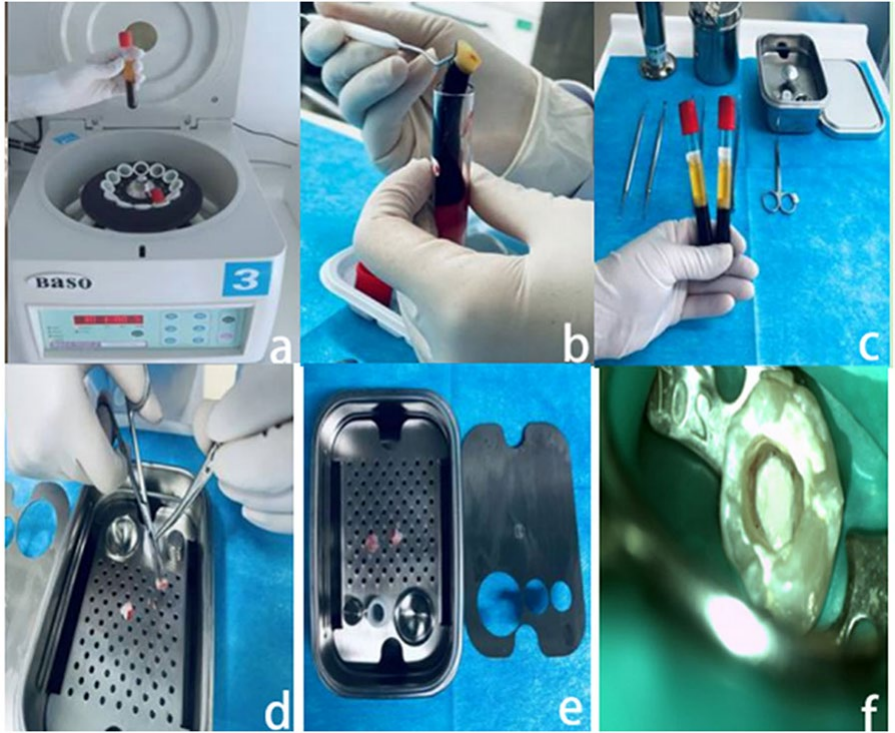
PRF assisted mature permanent teeth revascularization operation diagram. **a** Centrifugal treatment; **b** Preparation of PRF: After collecting blood and centrifuging, let it stand for stratification, and take the middle Layer PRF gel; **C** Take the supernatant; **d** Put the PRF membrane into the root canal; **e** Tooth with PDF film implanted; **f** Closed crown

The experimental group used PRF to assist mature permanent teeth revascularization treatment: (1) At the first visit (Root canal sealing): perfect X-ray, blood routine, and coagulation function examination before operation. Use articaine to local anesthetize the patient’s oral cavity, expose the decayed pulp and uncover the crown with a rubber dam, and construct a crown approach. Use 20 ml of 1.25% NaOCI to wash the root canal repeatedly for 5–10 min, then rinse the root canal with normal saline for 5 min, and then dry the root canal. Ciprofloxacin, metronidazole and minocycline powder were mixed at a ratio of 1:1:1, and saline was added to prepare a 0.1 g/L triple antibiotic paste. Seal the triple antibiotic paste into the root canal, use a conveyor to feed the catheter, cover the mouth of the root canal with a sterile cotton ball, and temporarily seal the cavity with a glass ionomer cement. The patient will follow up within 3–4 weeks after surgery. The paste was removed one week later. If the gums are swollen, painful percussion, etc., root canal disinfection and sealing medicine should be repeated until the patient’s teeth are healed. (2) At the second visit (PRF implement): Before drawing blood, confirm that the patient has no symptoms such as red and swollen gums, and the examination has no positive characteristics. Local anesthetize the patient’s oral cavity, remove the temporary sealing material, rinse the root canal with 20 ml of 17% ethylenediamine tetraacetic acid, and dry it with absorbent paper. Remove 5 ml of the patient’s median venous blood and centrifuge. After centrifugation (see Fig. 1a), the middle layer of PRF gel (see Fig. 1b) is taken out, take the supernatant (see Fig. 1c), taken out with sterile tweezers, and the gel is squeezed with sterile gauze to obtain a PRF film. Perform X-ray examination to detect the length of the patient's tooth root. Use a sterile 40# root canal file to puncture the root canal tissue beyond the root tip tissue 3-5 mm to allow blood to flow into the root canal. After that, the PRF membrane was cut into pieces and placed in the root canal (see Fig. 1d). iRoot BP Plus (Innovative Bioceramix Inc., Vancouver, Canada) was placed 4 mm below the enamel bone boundary and no more than 1-2 mm below the enamel cementum boundary (see Fig. 1e). A wet cotton ball was placed above the iRoot BP Plus, and the cavity was temporarily sealed with a glass ionomer cement (see Fig. 1f). X-rays were taken in parallel after operation. Close the crown and review the patient's constant pressure. One day after the operation, the glass ionomer cement was taken out, the hardness of the iRoot BP Plus was checked, and permanent filling was performed with light-cured resin. The patient will be reviewed 3–6 months after surgery.

The control group was treated with classic revascularization: the root canal sealing was the same as the experimental group. The root apical hole was pierced, and the blood was drawn so that the blood reached 4 mm below the border of the enamel bone. No PRF was placed in the root canal. The other steps were the same as the experimental group.

### Efficacy evaluation criteria

At six months after the operation, the patient had no symptoms such as pain, red and swollen gums, tooth tapping pain and no pain, no sinus in the gums, loose teeth consistent with normal teeth. X-ray examination of the root apex periodontal disease disappeared, the apex was gathered, the root canal cavity was reduced, and the root was extended. It is judged to be remarkable effect.

The patient has no symptoms such as pain, red and swollen gums, tooth percussion and no pain, no sinus in the gums, loose teeth consistent with normal teeth. X-ray examination of the root apex periodontal disease disappeared, and the root can not be continued. It is judged to be effective.

The patient has gum swelling and pain, hot and cold tingling, etc., gums have sinus. X-ray film shows the existence of apical periodontal disease, and the root can not be extended. It is judged to be invalid [13].

### Observation indicators

(1) The root improvement (root canal wall thickness and crown root length) was recorded 6 months after operation. The root canal wall thickness and crown root length were within the normal range. The greater the root canal wall thickness, the shorter the crown root length, indicating the better effect of the treatment. (2) the improvement of tooth function and patient satisfaction score were recorded.

### Sample size calculation

In the initial pretrial, three patients who needed mature permanent tooth treatment adopted autologous PRF technology, and 100% achieved effective results after three months. In the same period, 3 patients received treatment without autologous PRF technology, and only 1 patient achieved effective results after three months. The required sample size was calculated based on a two-tailed significance level of 0.05 and a statistical power of 0.8, resulting in a minimum sample size of 6 in each group. The present study protocol further referenced a recently published systematic review related to autologous platelet concentrates for regenerative endodontic treatment [14]. In the included studies, the sample size of each arm was approximately 5 to 15. Therefore, we selected 10 samples from each group in this study.

### Statistical methods

The data was analyzed by SPSS18.0 statistical software, the measurement data was described by ($\overline{x} \pm s$), and the comparison was performed by t test; the count data was described by percentage (%), and the comparison was performed by χ2 test. *P* < 0.05 indicated that the difference was statistically significant.

## Results

### Comparison of efficacy between the two groups

**Fig. 2:**
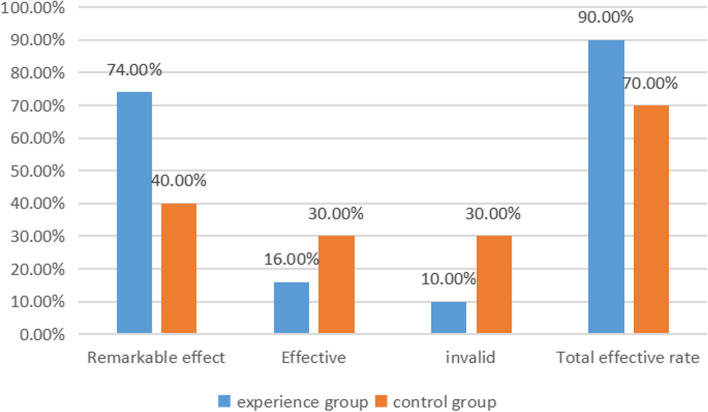
Comparison of efficacy between the two groups

After treatment, the total effective rate of the experimental group (100.00%) was higher than that of the control group (50.00%), and the difference was statistically significant (*P* < 0.05) (see Table 2 and Fig. 2).

**Table 2:** Comparison of efficacy between the two groups [n (%)]

group	n	Remarkable effect	Effective	invalid	Total effective rate
experience group	10	7 (70.00)	3 (30.00)	0 (0.00)	10(100.00)
control group	10	4 (40.00)	1 (10.00)	5 (50.00)	5(50.00)
*x*^*2*^					4.267
*P*					0.039

### Root canal wall thickness and crown root length before and after surgery

After the operation, the thickness of the root canal wall of the experimental group was higher than that of the control group, the length of the crown root was lower than that of the control group, and the difference was statistically significant (*P* < 0.05) (see Table 3).

**Table 3:** Root canal wall thickness and crown root length before and after surgery ($\overline{x} \pm s$,mm)

group	n	Root canal wall thickness		Crown root length	
		Before treatment	After treatment	Before treatment	After treatment
control group	10	2.08 ± 0.58	2.10 ± 0.38	0.69 ± 0.20	0.97 ± 0.31
experience group	10	2.05 ± 0.49	2.69 ± 0.73	0.62 ± 0.29	0.69 ± 0.27
*t*	-	0.125	2.267	0.628	2.154
*P*	-	0.902	0.036	0.538	0.045

### Patient's dental function and satisfaction score

**Fig. 3:**
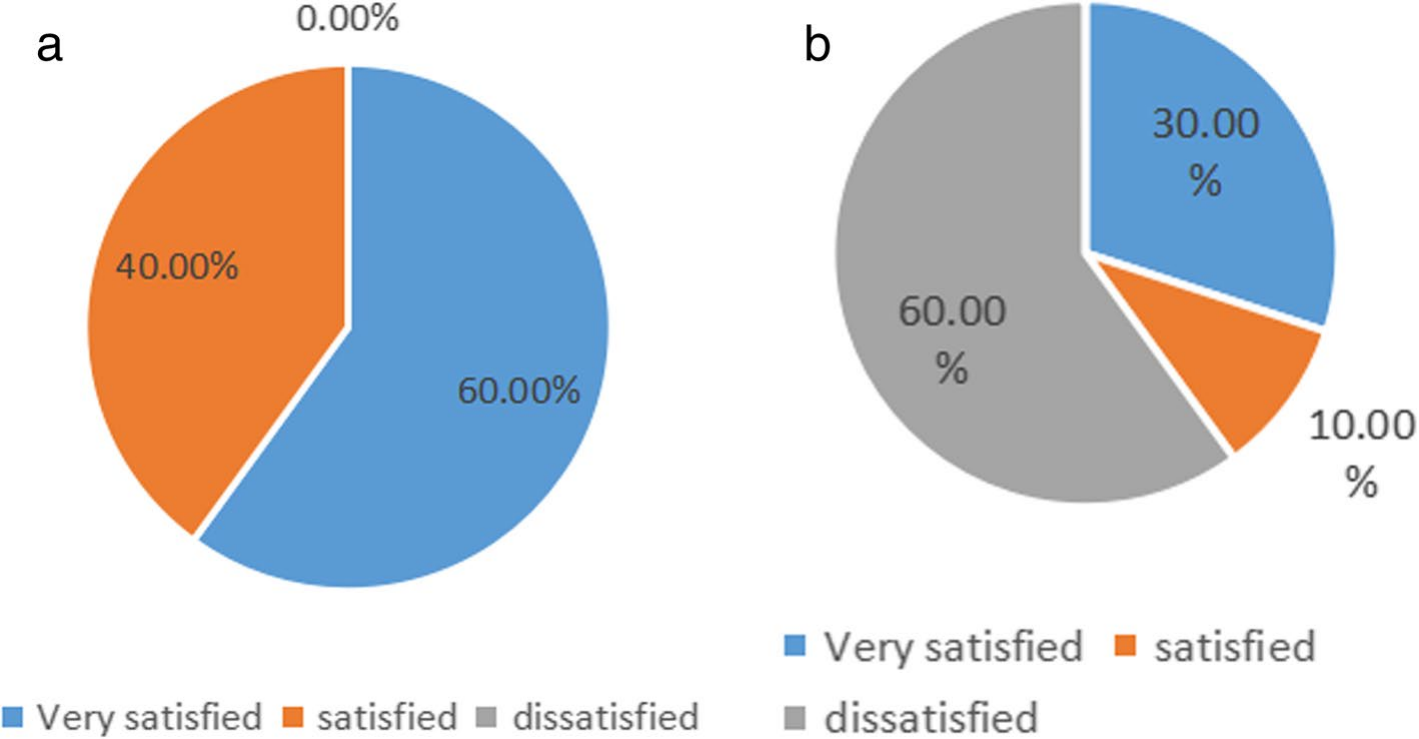
Comparison of satisfaction between the two groups (%). **a** Experimental group; **b** Control group

**Fig. 4:**
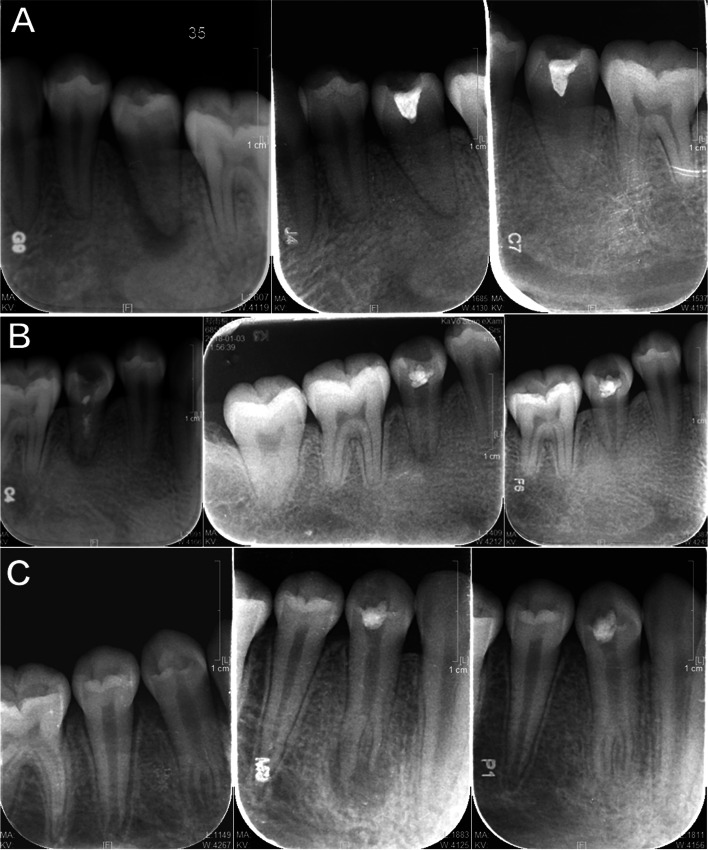
X-ray radiography showed the results of three cases in the experimental group (**A**-**C**, one case in each row) before treatment (left column), six months after treatment (median column), and two years after treatment (right column)

After the operation, the teeth occlusion, chewing function, color and overall aesthetic scores of the experimental group were higher than those of the control group, and the satisfaction rate of the experimental group was higher than that of the control group. The difference was statistically significant (*P* < 0.05) (see Tables 4, 5, and Fig. 3a, b). X-ray radiography also showed the results of three cases in the experimental group (Fig. 4, a-c).

**Table 4:** Comparison of dental function of patients ($\overline{x} \pm s$, points)

group	n	Occlusal degree	Masticatory function	color and lustre	Overall beauty
experience group	10	8.60 ± 1.20	8.90 ± 0.80	8.70 ± 0.90	8.50 ± 1.20
control group	10	7.50 ± 1.10	6.50 ± 0.40	7.60 ± 0.50	7.20 ± 0.90
*t*	-	2.137	8.485	3.379	2.741
*P*	-	0.047	0.002	0.003	0.013

**Table 5:** Comparison of patient satisfaction scores [n (%)]

group	n	Very satisfied	satisfied	dissatisfied	Satisfaction
experience group	10	6 (60.00)	4 (40.00)	0 (0.00)	10(100.00)
control group	10	3 (30.00)	1 (10.00)	6 (60.00)	4(40.00)
× *2*					5.952
*P*					0.015

## Discussion

Pulp revascularization can sterilize the tooth root, transform the necrotic pulp tissue into a sterile matrix, then stimulate the root tip bleeding, form a blood clot in the root canal, and generate pulp-like tissue to promote the continued development of the tooth root and improve the crown. Root ratio, to improve the strength of tooth roots, presents a better application prospect [15, 16]. PRF can assist in the application of pulp revascularization of mature permanent teeth, and the effect is good [17].

Pulp revascularization can protect the liver cells and active tissues around the roots of the patient's teeth, introduce the patient's own blood, form a biological scaffold, and promote the generation of tissues similar to the pulp [18]. This tissue has the sensation and function of normal pulp, which can enable mature permanent teeth to continue to develop, and eventually reach a level close to that of normal teeth, which is conducive to improving the hardness of the teeth, the thickness of the root canal wall, and the length of the root [19, 20]. Wu Tiantian [21] pointed out in the research that PRF is derived from the body, and the joint action of various components in inflammation regulation, angiogenesis, soft and hard tissue repair and regeneration and other physiological processes play important functions, and it has been gradually applied to young people. Permanent tooth pulp regeneration, apical barrier, delayed replantation and vital pulp preservation treatments, and the effect is good. Relevant data show that [22], pulp revascularization can repair infected or necrotic pulp, allow tooth roots to grow and develop, improve crown-to-root ratio, and increase root strength. The results of this study showed that the total effective rate of the experimental group (100.00%) was higher than that of the control group (50.00%), the thickness of the root canal wall of the experimental group was higher than that of the control group, and the crown root length was lower than that of the control group (*P* < 0.05). After the treatment, the thickness of the root canal wall and the length of the crown root have been improved, and most of the patients have achieved good results. This indicates that pulp revascularization promotes the continued development of the tooth root and accelerates the restoration of normal function of the tooth root. The reason is that, on the one hand, PRF provides a good root canal stent, providing sufficient space to store the hard tissue deposits on the inner wall of the root canal; on the other hand, PRF is rich in active factors, including cell chemokines, which promote cell entry and thereby Promote the restoration of dental pulp tissue [23, 24]. This is consistent with the research results of He X [25], which further confirms that PRF can provide a good scaffold material for pulp regeneration and the effect of pulp restoration is ideal. After treatment, the bite degree, chewing function, color, overall aesthetic score, and satisfaction of the experimental group were higher than those of the control group. Zhang Xin and others [26] selected 62 children with pulp necrosis as the research object. The control group underwent conventional pulp revascularization, and the observation group received PRF during the pulp revascularization. The total success rate of the observation group was 96.77%, which is significantly higher than 74.19% in the control group (*P* < 0.05). It is concluded that the application of PRF to young permanent teeth during pulp revascularization can improve the total success rate of treatment, postoperative root length and root canal wall thickness. The effect is better than that of conventional pulp revascularization surgery.

PRF is a fillable fibrin complex composed of platelets, cytokines and white blood cells. Compared to platelet-rich plasma, PRF is more economical and easier to prepare and is feasible in clinical practice [27]. Due to the great potential of PRF in clinical application. Its related technology is also constantly improving [28]. By adjusting the centrifugation procedure, injectable platelet rich fibrin (I-PRF) can be prepared without the use of anticoagulants. I-PRF has a three-dimensional fibrin meshwork while retaining the fluid nature, which has higher antibacterial, anti-inflammatory and regeneration abilities [29, 30]. With reference to the preparation protocol of I-PRF, higher concentrations of platelets and leukocytes were obtained from the buffy coat layer by high-speed centrifugation, which was named concentrated PRF (C-PRF). The growth factor release from C-PRF was then significantly increased and showed greater potential for cell migration and proliferation [31]. According to the "Low-Speed Centrifugation Concept", the preparation of PRF was further modified. An important product is Advanced-PRF (A-PFR), which leads to an increase in the number and distribution of platelets and leukocytes in the fibrin meshwork [32]. A-PRF is a variant of standard PRF that contains more growth factors with better regeneration potential and is commonly used in periodontal regeneration and implant surgery. The abovementioned materials provide a variety of therapeutic materials for dental pulp revascularization.

### Limitations

There are limitations in the literature. The evaluation indicators of this study are still less, and more indicators, especially quantitative results based on radiological tests, are still needed. This study was performed in a single center. Due to the differences in medical technology and equipment conditions in different hospitals, a multicenter study is needed to confirm the effectiveness of autologous PRF technology.

### Recommendations for future

Although this study confirmed the effectiveness of autologous PFR, PRF was still a complex mixture of multiple cytokines, growth factors, platelets, and various white blood cells. Furthermore, it is necessary to identify the major components that are beneficial for pulp revascularization based on omics research. The concentration and content of such beneficial components can be increased by adding exogenous active components, molecular ultrafiltration, etc., to further improve the therapeutic effect.

## Conclusion

In summary, autologous platelet-rich fibrin assists in revascularization of mature permanent teeth, can achieve ideal results, promote pulp regeneration, and can maximize the thickness of the root canal wall and crown root length within the normal range, and improve the treatment effect. It is worthy of further clinical promotion.

## Data Availability

The datasets used or analysed during the current study are available from the corresponding author on reasonable request.
